# Feline Calicivirus Infection: Current Understanding and Implications for Control Strategies

**DOI:** 10.3390/ani15142009

**Published:** 2025-07-08

**Authors:** Federica Di Profio, Matteo Carnevale, Fulvio Marsilio, Francesco Pellegrini, Vito Martella, Barbara Di Martino, Vittorio Sarchese

**Affiliations:** 1 Department of Veterinary Medicine, Università degli Studi di Teramo, Località Piano D’Accio, 64100 Teramo, Italy; fmarsilio@unite.it (F.M.); bdimartino@unite.it (B.D.M.); vsarchese@unite.it (V.S.); 2 Independent Researcher, 86082 Isernia, Italy; crn.matteo@gmail.com; 3 Department of Veterinary Medicine, Università Aldo Moro di Bari, S.p. per Casamassima Km3, Valenzano, 70010 Bari, Italy; francesco.pellegrini@uniba.it (F.P.); vito.martella@uniba.it (V.M.); 4 Department of Pharmacology and Toxicology, University of Veterinary Medicine, 1078 Budapest, Hungary

**Keywords:** feline calicivirus, variability, disease control

## Abstract

Feline calicivirus (FCV) is a highly contagious pathogen widely circulating in cat populations. FCV has been shown to be able to evade the host immune response through different mechanisms. As a result, following the acute phase of infection, some cats remain persistently infected or experience reinfection cycles with variants of the same strain or with distinct field FCVs. These animals may become asymptomatic carriers, assuming a critical role in virus transmission and posing a significant risk to susceptible cats, particularly in high-density settings. Typical clinical signs of FCV infection include upper respiratory tract disease, oral ulcerations, salivation, and gingivostomatitis. In some cases, FCV infection has also been linked to a range of other clinical manifestations, including severe virulent systemic disease with high mortality rates. Indeed, FCV diversity and evolution have led to the emergence of new genetic, antigenic, and phenotypic variants, challenging disease control. This review provides a comprehensive synthesis of FCV, including its molecular biology, epidemiology, pathogenesis and clinical manifestations. Additionally, the role of vaccination and direct prophylaxis is critically evaluated. An integrated approach is essential to mitigate FCV transmission and disease burden in feline populations.

## Simple Summary

Feline calicivirus (FCV) is a widespread and highly transmissible pathogen in cats, associated with a broad range of clinical presentations, from mild upper respiratory signs to severe and sometimes fatal systemic disease. The virus can also persist in healthy carriers and spread easily, especially in multi-cat environments. FCV high mutation rate contributes to the emergence of different strains, including highly virulent variants, making it harder to diagnose and control. This review outlines current knowledge on FCV, from its biology and patterns of transmission to the associated clinical presentations. The role of vaccination and other preventive measures is also discussed, highlighting the importance of a comprehensive strategy to manage and reduce FCV infections in feline populations.

## 1. Introduction

Feline calicivirus (FCV) is one of the most common and clinically relevant viral pathogens in cats. FCV is highly contagious and widely distributed within the general feline population [1]. Although primarily described as a feline respiratory pathogen, inducing oral and upper respiratory tract disease (URTD) [2,3], FCV infection has also been linked to a range of other clinical manifestations. Indeed, FCV, as a highly mutagenic RNA virus, exhibits significant genetic, antigenic, and phenotypic variability, resulting from ongoing evolution driven by point mutations, persistent infections, and recombination [4,5,6,7,8]. This variability has led to the emergence of various viral pathotypes with distinct tissue tropisms and levels of virulence, associated with different clinical diseases, including acute febrile lameness syndrome [9,10], dermatitis [11], abortion [12,13], severe pneumonia [14,15], acute enteritis [16] and the highly contagious and often fatal virulent systemic disease (VSD), characterised by a systemic inflammatory response syndrome [17]. Notably, the high genetic plasticity of FCV is both a consequence and a driver of persistent infection, as prolonged replication within hosts facilitates mutation and recombination, while antigenic diversification aids immune evasion and sustains long-term infection [6,7]. Given FCV complex evolutionary dynamics, impacting clinical relevance and also raising questions about the efficacy of current vaccines [18,19,20], this review aims to provide a comprehensive synthesis of current knowledge on its molecular evolution, epidemiology, pathogenesis, clinical manifestations and implications for disease control.

## 2. Aetiology

FCV is a member of the *Caliciviridae* family, which includes the genera *Vesivirus*, *Lagovirus*, *Norovirus*, *Sapovirus*, *Recovirus*, *Valovirus*, and *Nebovirus*, that infect mammals, *Bavovirus* and *Nacovirus,* that infect birds, and *Minovirus* and *Salovirus*, detected in fish. FCV belongs to the *Vesivirus* genus, along with other viruses that can cause vesicles on their host’s skin or mucous membranes [21].

**Figure 1:**
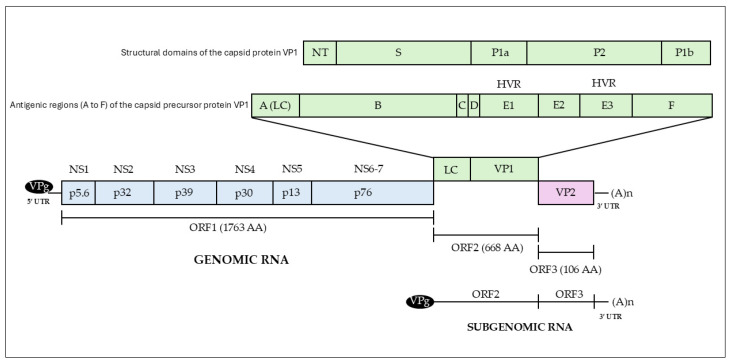
Genomic organisation of FCV genomic RNA with open reading frames (ORFs) 1 to 3, subgenomic RNA, antigenic regions (A to F) of the VP1 capsid precursor protein, and structural domains of the capsid protein VP1 [N-terminus (NT), shell (S), and protruding domain P with subdomains P1 (P1a and P1b) and P2]. LC, leader of capsid protein; HVR, hypervariable region.

Caliciviruses are non-enveloped viruses of 27–40 nm in diameter, with icosahedral symmetry, characterised by cup-shaped depressions on their surface to which they owe their name (from the Latin *calix*, meaning cup) [22]. The capsid encloses a single-stranded, positive-sense genomic RNA (g-RNA), ranging in size from 6.4 to 8.5 kb [21]. In addition, a subgenomic mRNA (sg-RNA) of about 2.2 to 2.6 kb and coterminal with the 3′ end of the g-RNA, is transcribed during infection [23,24,25,26]. Both the g-RNA and sg-RNA have a polyadenylated tail at their 3′ end. A 10–15 kDa virus protein genome-linked (VPg), covalently attached at the 5′ end, acts as a primer for genome replication and is responsible for initiating the translation of the viral RNAs [21,26]. The FCV viral genome (Figure 1) is about 7.7 kb long and is organised into three functional Open Reading Frames (ORFs).

ORF1 encodes a large polyprotein which undergoes post-translational autocatalytic cleavage to yield six non-structural proteins (NS1 to NS6/7) named, based on their molecular weight, p5.6, p32, p39, p30, p13, and p76. While p5.6, p32 and, p30 are still not well characterised, p39 is known as the putative NTPase, p13 is the VPg, and p76 is the proteinase-polymerase (Pro-Pol), a precursor of the FCV proteinase (3C-like protease) and the RNA-dependent RNA polymerase (RdRp) [27,28,29]. ORF2 encodes the capsid protein precursor (73 kDa), which is processed by the ORF1-encoded protease into the mature capsid protein (VP1) (~58 kDa) and the small ~14.7 kDa leader of the capsid (LC) protein, responsible for the cytopathic effect and the induction of apoptosis [27,30,31,32]. ORF 3, located at the 3′ end of the genome, encodes the ~12.2 kDa virus minor structural protein (VP2), which has an essential role in the maturation and assembly of infectious virions, as well as endosome escape [25,33,34].

The FCV capsid consists of 180 copies of the VP1, organised as 90 dimeric capsomeres assembled into a T = 3 icosahedral lattice, as well as 12 copies of the VP2 [21,35,36].

Each VP1 is functionally divided into the N-terminus (NT), the shell (S), and the C-terminal protruding (P) domains. The S domain forms a shell around the viral RNA genome, while the P domain, further divided into the P1 and P2 subdomains, is the most externally exposed. The P2 is located on the outermost face of the capsomere and is the site of the neutralising epitopes and the binding site for the cellular receptor, Feline Junctional Adhesion Molecule A (fJAM-A) [35,37]. Although fJAM-A is currently the only protein receptor identified for this virus family, it has been demonstrated that FCV can also bind to α2,3-linked sialic acid, a step crucial to FCV infection and likely involved in determining the tissue tropism of the virus [38].

Calicivirus replication likely proceeds through a minus-strand RNA intermediate used as the template for the synthesis of positive-sense full-length genomic and subgenomic RNAs [39]. The absence of a proofreading mechanism in the viral RNA polymerase makes the FCV genome prone to mutations during virus replication, contributing to the virus rapid evolutionary potential [40]. FCV exhibits one of the highest identified evolution rates among RNA viruses, ranging from 1.32 × 10^−2^ to 2.64 × 10^−2^ substitutions per nucleotide per year within the same host and from 3.84 × 10^−2^ to 4.56 × 10^−2^ substitutions per nucleotide per year within a population [7]. This rapid evolution contributes to the significant genetic heterogeneity observed among related isolates, suggesting that FCV exists within the host as a quasispecies, i.e., a mixed population of closely related sequences [4]. Based on amino acid sequence alignment and antigenic analysis, the capsid precursor protein is divided into six regions, from A to F [41]. Region A is cleaved into the LC protein during VP1 maturation, regions B, D, and F are highly conserved in FCV, whilst regions C and E are highly variable, exhibiting significant sequence divergence. Region E is further subdivided into 5′ and 3′ hypervariable regions (HVRs), separated by a conserved central domain. The 5′ HVR is a major antigenic determinant in FCV capsid as it contains the immunodominant neutralising epitopes of the virus [42,43].

Comparing the sequences of the VP1 5′ HVR has revealed that unrelated viruses typically show 20–40% genetic divergence, while isolates sharing a recent common ancestor generally differ by 5% or less. Genetic distances in the 5–20% range are uncommon [44].

## 3. Epidemiology

### 3.1. Transmission of FCV

FCV has a cosmopolitan distribution and is one of the most common viral pathogens of cats worldwide [45]. FCV typically causes high morbidity but low mortality in infected animals. The virus is highly contagious, and transmission occurs mainly through direct contact with an infected cat. FCV primarily spreads through oral and nasal secretions, but it has also been detected in ocular and rectal swabs, faeces, blood, and, occasionally, in urine samples [16,17,46,47,48,49,50,51]. Animals with acute infections are among the most important sources [1,48]. Due to FCV environmental resistance, indirect transmission can pose a significant risk, particularly in high-density conditions, like breeding facilities, catteries, and animal hospitals. Cages, litter trays, bowls, and cleaning tools can retain contaminated secretions and serve as fomites. Additionally, people who come into contact with infected cats, like owners and caregivers, can also act as passive carriers of the virus through contaminated hands, clothes, or medical instruments [17,52,53,54,55]. Although sneezing can disperse infectious droplets up to 1–2 m, aerosol transmission of FCV is considered limited, as cats do not typically emit significant infectious aerosols [56]. However, detection of viral RNA in ventilation filters suggests that aerosol spread cannot be entirely ruled out [55]. In addition, it has also been demonstrated that cat fleas (*Ctenocephalides felis*) can carry infectious FCV for up to 4 days and their faeces for up to 8 days at room temperature. In multi-cat settings, transmission may occur via ingestion during grooming, whilst flea bites do not seem to be a major route [57].

After recovering from the acute phase, some cats develop a persistent infection without apparent clinical signs, thus becoming asymptomatic carriers of the virus. While about half of cats with persistent FCV infections stop shedding the virus within 75 days, the infection can be lifelong in others. In carrier cats, the sites of virus persistence are the oropharynx and tonsillar epithelium, though tonsillectomy does not appear to successfully overcome the persistent infection. Shedding levels vary over time and between individuals due to multiple factors associated with host–virus interactions [47,48,58,59,60]. The mechanisms of FCV persistence are not fully understood, but immune-driven selective pressure promotes viral evolution, chiefly in VP1 regions E and C, allowing immune evasion. Persistently infected cats develop closely related quasispecies (>92% identity in region E) from the original strain [5,7]. Noteworthy, it was suggested that even live-attenuated vaccine strains may mutate and persist in cats [61,62,63]. Most long-term carriers undergo reinfection cycles, especially in high-density settings like shelters, where multiple strains circulate simultaneously, leading to cats being repeatedly infected with different variants of the same strain or with distinct field strains [5,7]. This phenomenon is particularly evident in settings with a high turnover rate of cats, resulting in frequent introductions of new FCV strains over time. Recombination events may also occur due to the infection of single cats with multiple strains, leading to even greater genetic variability [6,64]. This promotes the emergence of variants with differing virulence and pathogenicity [7,65], while stable households exhibit lower levels of FCV diversity [65].

### 3.2. Prevalence and Risk Factors

In domestic cats, the global prevalence of FCV can vary significantly over time between different countries and cat populations, and extensive research has been conducted to identify risk factors associated with the infection. Prevalence rates in healthy cats are generally low to moderate, ranging from 0% to 29% [66,67,68,69,70,71,72]. These rates increase in clinically diseased cats, ranging from 14.2% to 47% [2,66,67,68,73,74,75], and can reach up to 58% in cats with gingivostomatitis (GS) [69], who are found to be 8.3 times more likely to shed FCV than those without [8]. Infection rates are consistently higher in environments with dense cat populations compared to single-cat households or small groups of fewer than four cats [66,67,76,77]. In the study by Afonso et al. (2017), cats living in multi-cat households with 2–3 cats and 4–10 cats were, respectively, 1.7 and 2.8 times more likely to shed FCV than cats living alone [8]. Prevalence in shelters and cat colonies can vary widely, from as low as 0% to as high as 50–91% [5,78]. One study conducted in stray cat colonies in Northern Italy found a seroprevalence of 85%, indicating widespread virus circulation within the stray cat population [79].

The highest FCV detection rates were found in younger cats, especially those ≤ 12 months, likely due to their immune status [2,77,80], limited vaccination access, and maternal antibody interference [75]. As cats age, the risk of FCV infection decreases, particularly in the first 3–4 years [5,8,81]. However, some studies found no significant correlation between age and FCV risk of infection [68,75].

Sex is not a significant risk factor for FCV, and findings on the effect of reproductive status are inconsistent [8,67,75,76,77,82]. Some studies show a slightly higher FCV prevalence in males, possibly due to more aggressive behaviour in intact males [75,77]. Neutered cats may be less likely to test positive, possibly due to behavioural or hormonal changes affecting virus replication [8,67,77]. However, other studies found no significant correlation between the reproductive status and FCV positivity [76,82].

Finally, suboptimal hygienic conditions [66] and lack of vaccination [76] have been linked to higher infection rates, with unvaccinated cats being approximately 2 to 2.9 times more likely to be infected with FCV compared to vaccinated cats [75,81]. Nevertheless, vaccination does not provide complete sterilising immunity as it generally protects from developing severe clinical signs but does not prevent infection and the development of the carrier state. This explains why FCV remains prevalent in the general cat population despite widespread vaccination efforts [68,83].

### 3.3. FCV Host Range Beyond Domestic Cats

Cases of FCV infection have also been reported in other felines, such as European wildcats (*Felis silvestris silvestris*), tigers (*Panthera tigris*), lions (*Panthera leo*), and leopard cats (*Prionailurus bengalensis*), both in captive and wild settings [84,85,86,87,88]. An outbreak of feline herpesvirus and calicivirus in two black-footed cat (*Felis nigripes*) kittens and their dam was reported following the use of modified live virus vaccines in the kittens [89]. Highly virulent FCV strains were isolated from the faeces of a Siberian tiger in China and from an epizootic outbreak in captive exotic felids in the USA, both associated with mortality in naturally and experimentally infected animals [85,86]. Serological surveillance has detected the presence of FCV antibodies in wild felids worldwide, particularly in rural and urban areas where they may come into contact with feral and free-ranging domestic cats. These findings highlight the potential for viral transmission between domestic cats and wild felid populations, which may represent a threat to wildlife conservation and a source of highly virulent FCV strains for domestic cats [86,90,91,92,93,94,95,96,97].

Furthermore, there have been occasional reports of FCV isolated from dogs with glossitis and enteritis, likely infected through contact with cats [98,99,100,101,102,103]. In an Italian study by Di Martino et al. (2009), reporting the isolation of an FCV strain from the faecal sample of 3-month-old dog showing clinical signs of gastroenteritis, a serological investigation was also conducted on canine sera, revealing the presence of antibodies against the FCV-F9 vaccine strain, with a prevalence rate of 63.9% [103]. Additionally, Binns et al. (2000) observed a positive association between the presence of respiratory disease in dogs and the presence of FCV-infected cats within the same household [68], whilst Helps et al. (2005) reported a lower prevalence of FCV infection in catteries where dogs were present [66]. Despite these findings, the overall prevalence of FCV within the canine population and the specific role of dogs in FCV epidemiology remains poorly understood.

## 4. Pathogenesis

FCV enters the host primarily via the oro-nasal and conjunctival routes, binding to permissive cells through its VP1 protein [22]. The cellular receptor fJAM-A, a type I transmembrane glycoprotein, is found in endothelial and epithelial cells and regulates tight junction integrity and permeability [104]. Additionally, fJAM-A is also found on the surface of platelets, leukocytes, and erythrocytes, where it is involved in diapedesis and platelet aggregation [37,104,105]. FCV disrupts the homophilic interactions between fJAM-A molecules on adjacent cells, leading to compromised tight junctions and barrier integrity [105,106]. In cell culture, infected cells show a characteristic cytopathic effect associated with cell rounding and membrane blebbing [28]. FCV manipulates host cellular processes by inhibiting protein synthesis and inducing apoptosis. It selectively cleaves eukaryotic initiation factors (eIFs), disrupting host protein synthesis and redirecting the cell machinery toward viral protein production [107]. Apoptosis further aids viral spread by facilitating the release of viral progeny [108,109]. FCV varies in terms of tissue tropisms and pathogenicity. However, viral replication predominantly occurs in the oral, respiratory, and conjunctival tissues, where the lesions are generally limited in the case of less virulent strains. The most characteristic pathological feature of FCV infection is the development of oral vesicles that rupture over time, leading to necrosis of the overlying epithelium and the formation of ulcers. The ulcerated regions subsequently undergo significant neutrophil infiltration [1]. These lesions are caused by the disruption of epithelial cells tight junctions, which compromises tissue integrity [105]. However, since fJAM-A is widely distributed among different tissues, other factors likely influence the pathogenicity and tropism of different FCV strains [105], like their ability to also bind to α2,3-linked sialic acid [38]. The presence of the receptor in blood cells also enables the virus to cause a transient viraemic phase lasting up to 29 days [49]. Viremia typically begins 3–4 days post-infection, allowing the virus to disseminate to various organs and tissues following its initial replication in the oropharynx [110]. Different strains have been shown to affect the lungs, joints, intestines, and lower urinary tract, while some can cause severe systemic disease [1]. However, viremia is not exclusive to virulent systemic infections, as it has also been detected in cats exhibiting clinical signs confined to the upper respiratory tract [49]. Studies suggest that host factors, including age and immune status, influence disease severity. Older cats with virulent systemic infections exhibit more severe clinical signs, possibly due to an exaggerated immune response leading to more significant tissue damage [53,111].

## 5. Clinical Presentation

During FCV infection, a wide range of clinical signs and different syndromes can be observed (Table 1) depending on factors related either to the virus, such as tropism, virulence and infecting dose, or to the host, such as the cat’s overall health, age, immunity and genetic background. Husbandry factors may also play a role [1,22,110]. Concurrent infection with immunosuppressive viruses, such as feline parvovirus (FPV), feline immunodeficiency virus (FIV), and feline leukaemia virus (FeLV) can lead to more severe diseases [14,112,113,114]. In addition, the interactions with domestic cat hepadnavirus, highly common in cats [115,116,117,118,119,120,121,122,123,124] and with immunosuppressive potential [125] should also be considered.

### 5.1. Upper Respiratory Tract Disease

FCV is one of the main pathogens commonly associated with infectious feline upper respiratory tract disease (URTD), together with other viral agents, such as feline herpesvirus type 1 (FHV-1), and bacteria like *Bordetella bronchiseptica*, *Chlamydia felis* (*C. felis*), and *Mycoplasma* spp. [2,3]. Most FCV infections cause a mild, self-limiting syndrome, characterised by fever, oral ulcerations, and respiratory and conjunctival signs [67]. The onset of the disease is typically marked by depression and fever, with oral ulcers being the most characteristic clinical sign of FCV infection, typically on the tongue and palate, but occasionally also on the lips, nose, and rarely on other body parts. Cats may show anorexia and ptyalism due to oral ulcerative lesions [1]. Respiratory and ocular signs, such as sneezing and mild conjunctivitis, are less severe than in FHV-1 infections and are less common compared to oral lesions like stomatitis and gingivitis [67,68,80,126]. In field conditions, co-infections with FHV-1, *C. felis* and *Mycoplasma felis* are common [2,3,67,73,78] and might be responsible for more severe respiratory signs [15,67]. While mortality is generally low, some strains can cause fatal pneumonia, especially in cats co-infected with FHV-1 or other immunosuppressive viruses [14,15].

### 5.2. Feline Chronic Gingivostomatitis

Several studies have found a positive correlation between FCV and feline chronic gingivostomatitis (FCGS) [127,128,129,130,131,132], a debilitating condition affecting up to 26% of domestic cats [133]. A high percentage of cats with chronic stomatitis seem to be long-term carriers of FCV [134,135]. However, some research failed to find an association between FCV and FCGS, or to reproduce the disease through experimental infection, leading to ongoing debate due to inconsistent findings in the scientific literature [134,136,137]. Discrepancies in study outcomes may be attributed to differences in research methodologies, as variations in RT-PCR primers targeting poorly conserved regions of the FCV genome [132] or insufficient monitoring duration [134]. FCGS is characterised by erosive and/or proliferative inflammatory lesions, particularly in the regions lateral to the palatoglossal folds. Cats affected by this condition may experience moderate to severe oral pain, hypersalivation, reduced grooming, hyporexia, weight loss, irritability, withdrawn behaviour and decreased activity. The disease can persist for months or even years, significantly impacting the quality of life to the extent that some owners opt for humane euthanasia [138]. While FCV appears to be a significant factor in initiating FCGS, it is unlikely to be the sole causative agent.

### 5.3. Polyarthritis

FCV can occasionally cause polyarthritis, a condition known as limping syndrome [10,139], which is associated with lameness, joint pain, stiffness, hyperesthesia, and muscle soreness, often accompanied by fever, depression, and anorexia [9,10,140]. The association between FCV infection and lameness was clearly investigated in 1983, when Pedersen et al. [9] isolated two novel FCV strains, FCV-2280 and FCV-LLK, from the blood of kittens exhibiting these clinical signs [9]. Notably, some cats have presented with limping syndrome following vaccination with modified live FCV vaccines [141]. Lameness can be mild to severe, affecting one or multiple limbs, and frequently shifts between limbs within a short period [126]. Furthermore, it may develop concurrently with or following acute respiratory and oral symptoms, or as a prominent clinical sign [10,140]. In the affected joints, observed lesions include synovial membrane thickening, increased synovial fluid production, oedema and multifocal haemorrhages [10,139]. In most cases, full recovery occurs within 24–48 h without long-term effects on the joints [9]. In cases of persistent infection, lameness and joint lesions may still be observed several months after the onset of the disease [139]. The exact pathogenesis of limping syndrome is not fully understood. Several experimental infections, exploring the onset of the lameness pathotype and carried out using different FCV strains and different infection routes, showed controversial findings. In the study of Pedersen et al. [9] lameness was reproduced after oro-nasally inoculation of the virus, but joints resulted negative either for the presence of the virus or viral antigens [10]. Conversely, in a subsequent experiment FCV antigens were detected in the joints of experimentally inoculated cats [142]. However, either the intra-nasal infection with a respiratory strain or subcutaneous inoculation with the vaccine strain F9 were not able to induce lameness in unvaccinated or vaccinated animals [142]. Dawson et al. [10] isolated the virus from both normal and affected joints of specific pathogen-free cats infected either by intra-articular inoculation of a vaccine strain or by contact exposure to the F65 field strain originating from an outbreak of lameness [10]. Also, oro-nasal infection with a respiratory FCV 255 and a lameness-associated isolate FCV 2280 in kittens failed to induce severe upper respiratory signs, but both caused oral ulcers and lameness [126].

### 5.4. Virulent Systemic Disease

The most severe manifestation of FCV-induced disease is represented by the Virulent Systemic Disease (VSD), characterised by a haemorrhagic syndrome, multisystemic involvement, and high mortality [1]. However, it does not manifest as a well-defined syndrome with a clear clinical picture since it appears as a spectrum of possible clinical and pathological findings, primarily linked to systemic inflammation and vascular damage, driven by viral replication within vascular walls, leading to extensive vasculitis and multiorgan failure [111,143]. The incubation period of Virulent Systemic-FCV (VS-FCV) infection is usually 1 to 5 days, but it can sometimes extend up to 12 days. Although regularly vaccinated, adult cats over one year of age appear to be at higher risk of developing severe disease and mortality than kittens [53]. The mortality rate for VS-FCV ranges from 22 to 86%, depending on the outbreak [144]. Initial symptoms typically resemble a severe acute upper respiratory tract disease, characterised by sneezing, nasal congestion, and ocular discharge, along with hyperthermia, anorexia, lethargy, voice loss, and hyperirritability. As the disease progresses, pneumonia and pulmonary oedema may develop, causing severe respiratory distress. Varying degrees of diffuse cutaneous oedema, especially on the head and limbs, are characteristic and consistently observed. Dermatological manifestations are prominent, with ulcers extending from the oral cavity to the face, ear pinnae, and paws, accompanied by focal crusting, erythema, and hair loss around the nose, lips, eyes, and ears. Lesions may coalesce into larger, irregular crusts. Some cats appear jaundiced due to hepatic necrosis and liver failure [17,53,145]. In some cases, VS-FCV infection has been associated with thrombocytopenia and disseminated intravascular coagulation (DIC) [17]. High levels of circulating FCV may interfere with normal platelet function, potentially triggering platelet aggregation and secretion through interactions with fJAM-A. This mechanism could contribute to microthrombus formation, and the widespread vascular damage observed in affected cats [106]. Gastrointestinal signs such as vomiting and diarrhoea can also occur, along with joint oedema and lameness [17,53,145].

### 5.5. Paw and Mouth Disease

Interestingly, before the first reported outbreaks of VS-FCV in 1989, a similar syndrome named Paw and Mouth Disease (PMD), was described by Cooper and Sabine (1972) [146] in Australia. The affected cat showed oedema and pain in both right feet, along with ulcerative lesions on the paw pads, tongue, palate, lips, and pharynx, but maintaining good overall health, appetite, and recovered almost completely within a week of hospitalisation [146]. Few similar cases later reported in Australia [147] and Europe [11,148,149] were also characterised by fever, depression, anorexia, cutaneous oedema on the head and limbs, as well as ulcerative lesions on the paws, head and mouth. Fatal cases were rare, and they were either due to natural causes or euthanasia. Although the initial clinical presentation of the disease resembles that of VS-FCV infection, PMD differs from VSD as it generally lacks high morbidity and mortality, organ involvement and epizootic spread [11,148,149]. However, it remains unclear whether these cases represent mild forms of VSD or a distinct syndrome with a different clinical presentation [149].

### 5.6. Enteritis

Enteritis may also be considered a clinical manifestation associated with FCV infection. In fact, the first isolation of FCV has been documented from the gastrointestinal tract of cats [46], and a number of subsequent studies have supported this association. Experimental infections conducted by Povey and Hale (1974) induced diarrhoea in two specific pathogen-free cats, along with other typical clinical signs of FCV infection [150]. Mochizuki [151] isolated five strains of FCV from either normal or diarrhoeic stools of cats in Japan [151]. More recently, FCV RNA was detected in 35.7% (5/14) of diarrhoeic kittens during an outbreak of gastroenteritis in a shelter in the USA [152] and in 9.6% of cats with enteritis in Japan [153]. In the study by Di Martino et al. [16] analysing faecal samples from cats with diarrhoea, FCV RNA was identified in 25.9% (62/239) of clinical cases and in 0% (0/58) of controls. FCV was the sole pathogen in 50% of positive animals [16]. It has been suggested that FCV may pass through the alimentary tract and be excreted in faeces after replicating in the respiratory tract. However, in vitro susceptibility evaluation showed that strains isolated from the gastrointestinal tract are more stable than those isolated from the upper respiratory tract when exposed to low pH, bile salts, and trypsin, supporting the classification of FCV into enteric (E-FCV) and respiratory (R-FCV) strains [16,151]. Overall, despite the detection of FCV in diarrhoeic samples, its role as a primary gastrointestinal pathogen or as a co-factor in feline enteric disease requires further investigation [16].

### 5.7. Feline Lower Urinary Tract Disease and Abortion

Additionally, FCV has been proposed as a contributing factor in feline lower urinary tract disease (FLUTD), particularly in idiopathic cases, supported by viral isolation from urine and urethral plugs in affected cats, though a direct causal role remains unconfirmed [51,154,155,156,157,158,159]. Finally, transplacental transmission and abortion were considered as potential consequences of FCV infection in pregnant queens with virus isolation from aborted foetuses [12,13].

## 6. Prevention and Control

### 6.1. Immunity and Vaccination

The widespread diffusion of FCV and the severity of associated syndromes make active immunisation an essential preventive measure to protect the feline population [160,161]. Despite the genetic and antigenic diversity of FCV strains, they are classified as a single but heterogeneous serotype, generally exhibiting an acceptable level of cross-reactivity [8,162,163]. The European ABCD vaccine recommendation for cats mentions that all cats should be vaccinated against FCV [164]. Indeed, according to the World Small Animal Veterinary Association (WSAVA) Guidelines [160], vaccination against FCV is included in the “core vaccines” set for cats, in association with vaccines protecting against FPV and FHV-1. FCV vaccines have been widely available and commonly used in the feline population [1]. Immunisation does not prevent infection but generally mitigates the severity of the disease, decreases viral loads, and the duration and extent of oropharyngeal shedding and FCV viremia [49,165]. However, unvaccinated cats face a significantly higher risk, being 2 to 2.9 times more likely to contract FCV infection than vaccinated ones [75,76,81]. Both modified live virus (MLV) and inactivated parenteral vaccines are available. Although the MLV vaccines induce strong cell-mediated and humoral immunity, they still retain some pathogenic potential and should be administered with caution [160]. Clinical signs can occur if the vaccine is accidentally aerosolised or spilled onto a cat’s skin and ingested during grooming [1,44,141]. Furthermore, they can occasionally replicate, causing disease in the vaccinated cat and transmission to other cats [28,61]. This can be especially true when MLVs are administered to non-domestic carnivores, as demonstrated by an outbreak of feline herpesvirus and calicivirus in black-footed cats (*Felis nigripes*) in a zoo following vaccination [89]. Finally, replication of FCV vaccine strains can contribute to the emergence of immune-evasive variants or potentially severe diseases in immunocompromised animals [166]. However, genetic analyses have shown that most FCV isolates involved in suspected vaccine reactions are field strains, with vaccine-derived viruses rarely detected in these cases or in the broader cat population [7,44,58,62,63]. Inactivated FCV vaccines are effective but require an adjuvant to enhance their immunogenicity and trigger an adequate immune response. However, adjuvants in inactivated vaccines have been linked to feline injection-site sarcoma (FISS) [167], prompting the development of non-adjuvanted inactivated vaccines [168].

Multiple FCV strains are used for the formulation of commercially available vaccines. FCV-F9, isolated in 1950, is currently the oldest and one of the most used modified-live strains [110]. Over time, this strain may have undergone minor genetic modifications due to viral evolution during cultivation [4,49,61]. The second available vaccine in Europe consists of an inactivated, non-adjuvanted double-strain formulation containing FCV 431 and FCV G1 [163,168,169]. Both formulations are often administered in combination with FHV-1 alone or alongside FHV-1 and FPV [170]. The inactivated FCV 255 strain was formerly included in a multivalent European vaccine with FHV-1, FPV, FeLV, and *C. felis*. Although this formulation is no longer available [22], FCV 255 remains widely used in some Asian countries [64,171]. In Japan and Korea, new inactivated vaccines based on local FCV strains have been developed, including trivalent and bivalent formulations [171,172]. In the USA, a non-core bivalent vaccine combines a standard strain with an isolate obtained from virulent systemic disease, although its efficacy against heterologous VS-FCV strains remains uncertain [173]. In the USA, intranasal modified-live FCV vaccines are still commercially available and able to induce a strong mucosal IgA immunity 2 to 4 days after a single administration [174,175]. They are less affected by maternally derived antibodies compared to parenteral alternatives [160]. However, intranasal administration may lead to temporary oro-nasal shedding of the vaccine virus and mild to moderate respiratory symptoms such as sneezing and oculo-nasal discharge [1]. Besides the currently available commercial vaccines, researchers have explored the development of other vaccine types, including protein subunit vaccines [176,177], virus-like particle (VLP) vaccines [178], recombinant feline herpesvirus-1 expressing an FCV capsid protein [179,180,181], and DNA vaccines [182]. These experimental vaccines successfully induced the production of neutralising antibodies against multiple FCV strains and provided varying degrees of protection. However, none have been licenced for commercial use [82].

Young kittens acquire passive immunity through maternally derived antibodies (MDAs), provided that the mother has acquired immunity before giving birth [183]. Vaccination should be avoided during pregnancy, but, if necessary, only inactivated vaccines are recommended [160]. MDAs are primarily transferred with colostrum, which contains trypsin inhibitors that prevent their degradation in the gastrointestinal tract. IgG is the main Immunoglobulin in colostrum and protects kittens during their early life [183]. However, the effectiveness and duration of MDA transfer vary among individuals and litters [184]. MDAs have an estimated half-life of 15 days and typically persist for 10–14 weeks [185,186,187]. However, 20% to 26% of kittens may lack detectable antibodies as early as six weeks of age [186]. This creates a vulnerable period in which MDAs may be too low to protect against infection but still high enough to interfere with vaccination, potentially leading to vaccine failure [188]. Without serological testing, it is not possible to accurately assess the kitten’s protection status or ability to respond to vaccination. The WSAVA Guidelines for the vaccination of dogs and cats [160] recommend starting core vaccinations for kittens at 6–8 weeks of age, with boosters every 2–4 weeks until they are at least 16 weeks old. However, the vaccination protocol should be chosen based on a risk assessment that considers the cat’s age, health status, lifestyle, housing conditions, and the associated risk of FCV infection [160,161]. Early vaccination is advised for kittens in high-risk environments, such as shelters [161,186]. Ultimately, the number of primary core vaccines administered depends on the kitten’s age at the start of vaccination and the selected intervals between them [160]. It is advisable to use the same vaccine brand or, at minimum, the same vaccine strain throughout the entire primary vaccination series [110]. A booster is typically given at 12 months of age or one year after the last dose [160,161]. Its main goal is to protect cats that failed to respond to the primary series, rather than merely boost immunity. Although the 12-month timing suitably aligns with the first annual health check, delaying this dose may leave non-responders vulnerable to infection. To reduce this risk, WSAVA guidelines suggest administering the booster as early as 26 weeks of age, or anytime between 26 and 52 weeks [160]. After the primary vaccination cycle, immunity can last 3–4 years [189,190], and booster doses every three years are advised for low-risk cats, mainly indoor-only or without contact with other animals, either for WASAVA [160] or ABCD vaccination recommendations [164]. However, it is important to make owners aware that protection against clinical disease tends to decline as time since the last vaccination increases [110]. In higher-risk settings, as catteries or multi-cat households, annual revaccination is generally recommended [160]. Older cats with an unknown FCV vaccination history should receive two doses 2 to 4 weeks apart to establish an adequate immune response, followed by a booster one year later, using vaccines with the same virus strains [110,160]. A semi-quantitative in-house test for the detection of FCV, FPV, and FHV-1 antibodies is available in several countries to determine whether vaccination is required during a cat health evaluation, although the benefit of measuring FCV antibodies before vaccination is controversial [191] also considering that antibodies detected in a cat do not necessarily protect against the strains in the field [110].

### 6.2. FCV Management

FCV control is particularly challenging in multi-cat environments. Effective strategies are multifactorial and focus primarily on limiting viral transmission within the population. Reducing group size, minimising new introductions, enforcing quarantine, and isolating symptomatic cats are essential [110]. Good hygiene protocols play a crucial role in FCV control, and the use of disinfectants with proven efficacy against FCV is necessary in environments such as shelters, boarding facilities, breeding colonies, cat shows, catteries, and veterinary hospitals. FCV can remain stable in the environment for several weeks, up to one month, and shows resistance to lipid solvents and numerous common disinfectants, such as quaternary ammonium compounds (QACs), phenol, and pine oil at standard disinfectant concentrations [192,193,194,195]. Alcohol’s effectiveness against FCV varies significantly based on the type and concentration. 1-propanol is the most effective both in vitro and in vivo [196]. Ethanol is less effective in vitro, but as effective as 1-propanol in vivo. 2-propanol is the least effective in both settings [196]. Alcohol-based sanitisers primarily inactivate the virus by denaturing capsid proteins, a process that requires water; therefore, middle-range alcohol concentrations (50–70%) are more effective than higher ones (90%) [196]. The pH can also significantly influence the efficacy of ready-to-use ethanol (60%), which was proven to be more effective at a short contact time when formulated at higher alkaline pH between 10.8 and 12.0 [194]. Effective virucides (Table 2) against FCV include oxidising agents like sodium hypochlorite (bleach) at a concentration of 5400 ppm with a 1 min contact time or for at least 5 min at a concentration of 2700 or 1350 ppm, and accelerated hydrogen peroxide (AHP) at 7000 ppm with a 5 min contact time, or at 3500 ppm in 10 min [192]. Chlorine dioxide at a concentration of 10 ppm and 1% potassium peroxymonosulfate, both with a 10 min contact time, have shown virucidal effect against FCV while being less corrosive for steel and good-quality medical instruments [193]. Other effective oxidising agents include copper iodide (CuI) nanoparticles and Ozone (O_3_), with efficacy varying based on dose and contact duration [197,198]. Sodium bicarbonate alone at 5% concentration or combined with glutaraldehyde or activated dialdehyde also showed an effective virucidal effect in a short contact time. This is an inexpensive and non-toxic disinfectant for both cats and humans; however, it is not effective against numerous other pathogens [199]. Besides the chemical compound used and the exposure time, the effectiveness of disinfection is also strongly influenced by organic impurities [192,200] and the intrinsic resistance of FCV strain. Many commercial disinfectants approved for calicivirus inactivation and commonly employed against FCV have been tested using the laboratory-adapted F9 vaccine strain. Nevertheless, due to its genetic and antigenic diversity, various FCV strains have shown differences in their environmental stability and resistance to pH variation and disinfectants, with field strains generally being more resistant to inactivation than vaccine strains [201,202,203].

Interestingly, in recent years, in light of the threat posed by antimicrobial resistance and the need for alternative therapeutic strategies, essential oils (EOs) have gathered increasing interest as alternative antiviral agents. Their application has been investigated against various viral pathogens, including FCV, which is studied primarily as a surrogate model for non-enveloped viruses, such as human noroviruses (NoVs), particularly in research aimed at improving disinfection protocols and antiviral interventions in the food industry.

Several EO compounds have been tested in vitro for virucidal effects against FCV (Table 3). Notably, investigating the virucidal activity of clove, oregano, and zataria EOs against NoV surrogates, 2% oregano EO was able to decrease FCV titer [204]. Germacrone [205], *Artemisia princeps* var. *orientalis* EO and its main component *α-thujone*, investigated for their anti-FCV-F9 properties, also showed the ability to reduce the viral infectivity [206]. Pellegrini et al. [207] reported the virucidal efficacy of lemon EO [207] while a subsequent study reported no significant activity for *Thymus vulgaris* L. EO, *Rosmarinus officinalis* L. EO and *Salvia officinalis* L. EO at various concentrations and time contacts (10, 30 min, 1, 4 and 8 h) against FCV. In contrast, Melissa officinalis EO significantly decreased FCV titer compared to controls, although the effective concentration exceeded the cytotoxic limit of 123.02 μg/mL [208]. Overall, these findings suggest potential applications of EOs not only in environmental decontamination but also in veterinary settings. However, despite encouraging preliminary results, further studies are required to validate their antiviral efficacy, standardise concentrations, and evaluate safety and cytotoxicity in target species.

## 7. Discussion and Conclusions

The high degree of FCV genome plasticity contributes to the continuous emergence of new genetic, antigenic, and phenotypic variants, including the highly pathogenic systemic variants. Of particular clinical concern, there is a growing number of reports on VS-FCVs from VSD cases or outbreaks worldwide, including North America [53,54], Europe [52,145,209,210,211], Asia [212,213,214], and Australia [144], reaching mortality rates of up to 79%. In some studies, it has been suggested that these highly virulent strains emerge independently from genetically distinct FCVs [106,145,209]. In 2019, Brunet et al. [215] analysed the VP1 region E and proposed seven key amino acid positions (438, 440, 448, 453, 455, 465, and 492) as significant markers to differentiate VSD strains from classical respiratory FCVs. Nevertheless, overall efforts to identify consistent genetic markers that clearly distinguish FCV pathotypes have remained inconclusive [16,111,141,216,217,218]. Accordingly, virulence may depend on a combination of genetic and host immunological factors that are not yet fully understood. Accordingly, expanding sequence-based analyses to a broader set of FCV strains could provide new insights into viral pathogenesis and the molecular determinants of disease severity. The antigenic variability among FCV strains has been suggested to compromise the efficacy of current vaccines, particularly in the context of emerging virulent systemic variants [18,20,166]. While several studies report limited cross-neutralisation between vaccine strains (such as FCV-F9 and FCV-255) and circulating field isolates [11,17,19,53,54,168], other investigations demonstrate a broad cross-reactivity, with no significant antigenic or phylogenetic divergence observed among FCV-F9 strain and contemporary FCV isolates across Europe [8,219]. Given these conflicting findings, ongoing antigenic surveillance, based on genomic surveillance data to address the diverse and rapidly evolving strains of FCV, is warranted. Furthermore, it could be useful to routinely collect serum samples from both vaccinated and naturally infected cats, especially against genetically characterised circulating strains, assessing cross-reactive immunity. In addition, monovalent or polyvalent updated vaccines tailored to epidemiologically representative strains that are maximally cross-reactive against all circulating variants could appear promising. In conclusion, advancing our understanding of the epidemiology, genetic diversity, and evolutionary characteristics of circulating FCV strains is essential to address the ever-evolving landscape of FCV variants, with significant implications for infection control and the improvement of current vaccination strategies.

## Figures and Tables

**Table 1:** Clinical syndromes associated with FCV Infection.

Syndrome	Main Clinical Signs	Relevant Features
Upper respiratory tract disease	Sneezing, nasal discharge, conjunctivitis, oral ulcerations, depression, anorexia, ptyalism and fever.	Most common clinical presentation. Generally self-limiting but may predispose to secondary bacterial infections.
Limping syndrome	Lameness, joint pain, stiffness, hyperesthesia, muscle soreness, fever, depression, and anorexia. Previous or concurrent acute respiratory and/or oral symptoms.	Usually resolves within 24–48 h. Immune-mediated mechanisms are thought to contribute to pathogenesis.
Virulent systemic disease	Pyrexia, oedema on the head and limbs with or without lameness, oral ulceration, facial oedema, crusted lesions, ulcers, and alopecia on the nose, lips, ears, around the eyes, mouth, tongue and footpads. Less commonly, jaundice, gastrointestinal signs, bleeding and dyspnoea.	Epizootic spread with multisystemic involvement and high mortality. Caused by highly virulent FCV strains. Pathogenesis involves direct viral cytopathic effects on epithelial and endothelial cells, combined with immune-mediated responses.
Paw and mouth disease	Cutaneous oedema and ulcerative lesions on the skin of paws and on the head, in and around the mouth, and in the perianal region, as well as fever, depression and anorexia.	Uncommon; reported in single cases or in very small outbreaks, without an epizootic course. It may resemble virulent systemic disease.
Feline chronic gingivostomatitis	Moderate to severe oral pain, hypersalivation, reduced grooming, hyporexia, weight loss, irritability, withdrawn behaviour, decreased activity.	Chronic, multifactorial disease in which FCV is believed to be one of several contributing agents.
Enteritis	Diarrhoea.	FCV has also been detected in faecal samples from clinically healthy cats. Its role as a primary gastrointestinal pathogen remains to be fully elucidated.
Carrier state	No clinical signs.	Long-term viral shedding possible; significant role in transmission.

**Table 2:** Active compounds tested against FCV in vitro.

Compound	Concentration *	Contact Time	Reference
**1**-propanol	60%	30″	[196]
**2**-propanol	58%	1′	[196]
Ethanol	67%	1′	[196]
Chlorine dioxide	10 ppm	10′	[193]
Potassium peroxymonosulfate	1%	10′	[193]
CuI nanoparticles	1000 μgml^−1^	60′	[198]
O_3_	20–50 μg/mL	3′–5′	[197]
Sodium bicarbonate	5%	1′	[199]
Sodium hypochlorite	5400 ppm	1′	[196]
	2700 ppm	5′	
	1350 ppm	5′	
Accelerated hydrogen peroxide (AHP)	7000 ppm	5′	[196]
	3500 ppm	10′	

* Concentration that showed the highest virus-inactivating properties in experimental studies at a given contact time.

**Table 3:** Virucidal activity of essential oils against FCV.

Essential Oil (EO)/Compound	Concentration	Contact Time	Effect/Viral Titer Reduction	Reference
Oregano EO	2%	2 h at 37 °C	*↓ 3.75 log_10_ TCID_50_/mL	[204]
Germacrone (EO compound)	Dose-dependent	n/a	Inhibited replication in CRFK cells	[205]
*Artemisia princeps* EO	0.1%	1 h	~48% plaque reduction	[206]
*α-Thujone* (EO compound)	25 mM	1 h	↓ 1 log_10_ PFU/mL	[206]
Lemon EO	3020.00 μg/mL	8 h	↓ 1.25 log_10_ TCID_50_/50 μL	[207]
*Melissa officinalis* EO	12,302.70 μg/mL	10 min	↓ 0.75 log_10_ TCID_50_/50 μL	[208]

*↓ denotes a reduction in viral titer. Abbreviations: TCID, Tissue Culture Infectious Dose; CRFK, Crandell-Reese Feline kidney, PFU, Plaque Forming Unit.

## Data Availability

No new data were created.
